# Hydrogen Promotes the M1 Macrophage Conversion During the Polarization of Macrophages in Necrotizing Enterocolitis

**DOI:** 10.3389/fped.2021.710382

**Published:** 2021-11-17

**Authors:** Shenghua Yu, ZhiBao Lv, Zhimei Gao, Jingyi Shi, Qingfeng Sheng, Lulu Zheng, Junmei Zhou, Xueli Wang

**Affiliations:** ^1^Department of Pediatric Surgery, Shanghai Children's Hospital, Shanghai, China; ^2^Department of Center Laboratory, Shanghai Children's Hospital, Shanghai, China; ^3^Department of Critical Care Medicine, Shanghai Children's Hospital, Shanghai, China; ^4^Department of Pathology, Shanghai Children's Hospital, Shanghai, China

**Keywords:** necrotizing enterocolitis, macrophage polarization, hydrogen molecule, NF-κB, mice

## Abstract

**Background:** Hydrogen is protective against intestinal injury in necrotizing enterocolitis (NEC), mainly through to alleviate inflammation response. The M1 macrophages can promote inflammation. We hypothesized that hydrogen would promote the M1 macrophages conversion during the polarization and reduce the inflammatory factors in NEC.

**Methods:** We used M1 and M2 macrophages induced from RAW264.7 cells and bone marrow-derived macrophages, models of NEC and macrophages derived from spleens, abdominal lymph nodes and lamina propria in model mice. Cytokines, CD16/32 and CD206 were measured by quantitative PCR, flow cytometry. Nuclear factor-κB (NF-κB) p65 were determined by western blot. Histology staining were used to assess the severity of NEC.

**Results:** Macrophages were successfully polarized to M1 or M2 by assessing the expression of inflammatory factors. Pro-inflammatory factors and CD16/32 in M1 macrophages were decreased, and the expression of CD16/32 in lamina propria were inhibited after treatment with hydrogen, but the changes has no effects in other tissues. Hydrogen inhibited the NF-κB p65 in M1 macrophages nucleus and distal ileum of NEC. HE staining showed hydrogen could attenuate the severity of NEC.

**Conclusion:** Hydrogen could attenuate the severity of NEC through promoting M1 macrophages conversion by inhibited the expression of NF-κB p65 in the nucleus.

## Introduction

Necrotizing enterocolitis (NEC) is a serious gastrointestinal disease, mainly affecting premature neonates, especially in extremely low-birth-weight infants. Despite over six decades of clinical and basic research, the pathogenesis of NEC remains unclear (1). The overall mortality rate associated with NEC is between 20 and 30% (2, 3). Premature infants have immature intestinal and deficient immune function. Some scholars expressed that insufficient immune function is involved in the pathogenesis of NEC, and macrophages play a vital role in the development of NEC as well (4–7).

Macrophages play an important role in the development, progression, and resolution of inflammation disease (8). Two major subtypes of polarized macrophages are called classical macrophages (M1) and alternative macrophages (M2). Activated M1 macrophages produce pro-inflammatory cytokines, such as interleukin 6 (IL-6), IL-1β, nitric oxide, and tumor necrosis factor-α (TNF-α), and then contribute to inflammatory reaction. In contrast, M2 macrophages produce anti-inflammatory factors, including IL-10, tumor necrosis factor-β (TNF-β), Arg-1, and protein YM-1, which mainly promote tissue remodeling and anti-inflammatory response (8–11). Previous studies have shown that macrophages are plastic cells and M1 or M2 macrophages can alter their functional profiles in response to the micro-environmental changes (12). Wei et al. investigated the role of macrophages in murine model of NEC and showed that M1 macrophages can promote NEC by increasing intestinal epithelial apoptosis (13). Moreover, polarization of M2 macrophages has been reported to stimulate the ability of mesenchymal stem cells to ameliorate acute kidney injury in metabolic endotoxemia and protect the lungs against acute lung injury (14).

Molecular hydrogen as a medical gas to measure the local blood flow firstly reported in 1964, and then hydrogen was used as a biomarker for the early diagnosis of NEC (15, 16). Ohsawa et al. demonstrated that hydrogen can protect against oxidative damage by selectively reducing cytotoxic oxygen radicals, and our previous study also revealed that hydrogen-rich saline protects NEC from oxidative stress and inflammation (17, 18). It is well-known that NF-κB plays a key role in the immune system and polarization of macrophages (19–21). Xu et al. showed that inhalation of hydrogen gas protects liver against ischemia/reperfusion injury and also attenuates renal injury in severe acute pancreatitis and ameliorates bowel injury in a model of NEC by the NF-κB signaling pathway (22–24).

The aim of this study was to examine that the regulation of hydrogen on inflammatory factors in macrophages and mice model, and the role of NF-κB in M1 macrophages conversion, the then effect of hydrogen on macrophages in newborn mice of NEC.

## Methods

### RAW264.7 Macrophage Cell Culture

RAW264.7 macrophages were purchased from the Cell Bank of the Type Culture Collection of Chinese Academy of Sciences (Shanghai, China). Cells were grown in Dulbecco's modified Eagle's medium (DMEM) containing 10% fetal bovine serum (FBS; Gibco) and cultured at 37°C with 5% CO_2_ in a humidified incubator.

### Born Marrow-Derived Macrophages

Bone marrows were harvested from femurs and tibia of C57B6/J mice (aged 8–12 weeks) and cultured in the presence of recombinant mouse M-CSF (20 ng/ml; R&D Systems) in RPMI 1640 medium for 7 days as described previously (21, 25). BMDMs were washed and cultured in different mediums to polarize M1 or M2 macrophages.

### Animal Experiments

The following experimental protocols were performed according to the Ethical Guidelines for the Use of Animals in Research, and this study was approved by the Animal Care Committee of the Children's Hospital of Shanghai. In addition, 8-day-old C57BL/6J mice were used to establish the model of NEC as previously described (26–28). The mice were purchased from Shanghai Super B&K Laboratory Animal Corp. (Shanghai, China), and randomized into the following groups: Group 1, CON (*n* = 18); Group 2, NEC (*n* = 19); Group 3, CON + H_2_ (*n* = 18); and Group 4, NEC + H_2_ (*n* = 20). All the mice were hand-fed trans-orally every 4–5 h with artificial formula using a 24-G catheter. Groups 2 and 4 had asphyxia for 10 min exposure to 95% nitrogen, followed by daily cold exposure at 4°C for 10 min twice before feeding. Groups 3 and 4 were daily treated with hydrogen-rich water (20 ml/kg/day, 0.5 mmol/L) twice before stress. Groups 1 and 2 were fed with cleaned water as control. We fed model mice water in the last 12 h to remove the resident in the intestine of mice.

### Preparation and Stimulation of Macrophage Conditioned Medium

RAW264.7 macrophages and BMDM were polarized to M1 and M2 macrophages as described previously (25, 29). The following additives were added to the culture medium: (1) CON: no additional additive to maintain M0 macrophages; (2) M1: 20 ng/ml LPS and 50 ng/ml interferon gamma (IFN-γ) to generate M1 macrophages; (3) M2: 20 ng/ml IL-4 to generate M2 macrophages; (4) CON + H_2_: 0.6 mmol/L molecular hydrogen in normal culture medium; (5) M1 + H_2_: 0.6 mmol/L molecular hydrogen + 20 ng/ml LPS and 50 ng/ml IFN-γ; (6) M2 + H_2_: 0.6 mmol/L molecular hydrogen + 20 ng/ml IL-4.

### Hydrogen Treatment

The preparation of hydrogen-rich water was performed as described previously (18). Hydrogen was dissolved in water or DMEM medium for 4 h under high pressure (0.4 MPa) up to a supersaturation. The saturated hydrogen water or DMEM medium was sterilized by gamma radiation and stored under atmospheric pressure at 4°C. It was freshly produced every day and detected by a portable determination instrument ENH-1000 to ensure a stable concentration of 0.6 mmol/L (18, 30).

### Histology Staining and NEC Scoring

Distal ileum samples were first fixed in 4% formalin and embedded in paraffin, and then stained with hematoxylin and eosin (H&E). The histological slides were blindly evaluated by two independent observers using a published NEC scoring system. Grade 0, normal; grade 1, focal mild injury confined to villous tips; grade 2, partial loss of villi; grade 3, necrosis extending to submucosa; grade 4, transmural necrosis. NEC was here considered as grade 2 or above.

### Quantitative Reverse Transcription Polymerase Chain Reaction (RT-qPCR)

Total RNA was extracted from macrophages using TRIzol reagent (Catalog No. 15596-026; Life Technologies, USA) according to the manufacturer's protocol. Aliquots of 1 mg of total RNA were reversely transcribed using Super-Script Reverse Transcriptase (Invitrogen, USA) and Oligo-dT (18)-primers (Invitrogen, USA). The RT-qPCR was carried out by using SYBR Green Master Mix Kit in ABI Prism 7000 Sequence Detection System (Applied Biosystems, USA) according to the manufacturer's instructions. Glyceraldehyde-3-phosphate dehydrogenase (GAPDH) was amplified as an internal standard. The primers used are shown in Table 1. The expression levels of the messenger RNAs (mRNAs) were reported as fold changes vs. control.

**Table 1:** List of primers.

**Gene**	**Primer sequence (5'−3')**	**Product length**
IL-1β	(F) GCAACTGTTCCTGAACTCAACT	89 bp
	(R) ATCTTTTGGGGTCCGTCAACT	
iNOS	(F) CAGCTGGGCTGTACAAACCTT	310 bp
	(R) CATTGGAAGTGAAGCGTTTCG	
TNF-α	(F) CAGGAGGGAGAACAGAAA	68 bp
	(R) CCTGGTTGGCTGCTTGCTT	
Arg-1	(F) AGACAGCAGAGGAGGTGAAGAG	63 bp
	(R) CGAAGCAAGCCAAGGTTAAAGC	
YM-1	(F) GGATGGCTACACTGGAGAAA	178 bp
	(R) AGAAGGGTCACTCAGGATAA	
IL-10	(F) GCCGGGAAGACAATAACTGC	223 bp
	(R) GCCTGGGGCATCACTTCTAC	
GAPDH	(F) TGATGGGTGTGAACCACGAG	126 bp
	(R) GCCCTTCCACAATGCCAAAG	

### Flow Cytometry for Analysis of Macrophage Subtypes

M1 macrophages were identified with monoclonal antibodies specific for BB515-CD11b (BD, 564454) and APC-Cy7-CD16/32 (BD, 560541), and M2 macrophages were detected with antibodies specific for BB515-CD11b and Alexa Flour@647-CD206 (BD Biosciences, San Jose, CA, USA) antibodies, then CD16/32 or CD206 represent M1 or M2 macrophages, respectively. For immunophenotypic analysis, macrophages were gently detached with cold phosphate-buffered saline (PBS), pipetted into single cells, and then suspended at 1 × 10^6^ cells/ml. Cell suspensions were washed with PBS and then followed by incubation with the antibody mixtures for 30 min on ice boxes in the dark. Cells were then washed with buffer solution twice. Data were immediately acquired by using BD LSR II using FlowJo software.

To isolate the cells from the lamina propria of newborn mice with the method previously described (21, 31), 2-cm distal ileum samples were cleaned from mesentery, finely minced with scissors, and then incubated in DMEM containing 10% FBS and 10 mM dithioerythritol (D8255; Sigma-Aldrich, USA), which was preheated to 37°C. Tissue was incubated for 20 min with gentle agitation. Supernatants containing the enterocyte were discarded. Lamina propria leukocytes were then isolated from the remaining tissue digestion at 37°C for 40 min in RPMI, 10% FBS, 100 U ml^−1^ collagenase (C0130; Sigma-Aldrich, USA), and 15 μg/ml DNase (D4527; Sigma-Aldrich, USA) with gentle agitation. After that, cells were washed twice in cold PBS with 1% BSA and filtered through sequential 70-μm and 40-μm cell strainers.

### Western Blot Analysis

Distal ileum tissues were weighted and cut into small pieces according to the manufacturer's instructions. Macrophages were washed with PBS and collected for extracting proteins. Cytoplasmic and nuclear proteins were extracted using the EpiQuickTM Nuclear Extraction Kit I (OP-0002; EpiGentek, Farmingdale, NYUSA) (32). The nucleoprotein concentration was quantified with bicinchoninic acid (BCA) Assay Kit (Thermo Fisher Scientific, USA) and stored at −80°C. The protein samples were denatured at 100°C for 5 min, separated on 10% acrylamide gels, and then electrotransferred to polyvinylidene fluoride (PVDF) membranes. Afterwards, the membranes were blocked in TBST containing 5% fat free milk at room temperature for 2 h. The blots were incubated overnight at 4°C with primary antibodies: rabbit anti-mice P65 (ab32536; Abcam, UK) and Lamin B1 (12987-1-AP; Proteintech, USA) at 1:1,000 and 1:2,000, respectively. The membranes were washed three times with TBST and incubated for 1 h at room temperature with goat anti-rabbit IgG and goat anti-mouse IgG (1:5,000; Proteintech, USA). After washing three times for 10 min with TBST, the membranes were visualized and imaged using a chemiluminescent substrate (Clinx, Chemiscope 3500, China). Lamin B1 protein was used as an endogenous control as well.

### Statistical Analysis

Statistical analysis was performed using SPSS 22.0 software (IBM, USA), and the data were presented as mean ± standard deviation (SD) of at least three independent experiments. The incidence of NEC was compared with chi-squared test. A *p*-value < 0.05 was considered statistically significant.

## Results

### Effect of Hydrogen on Inflammation Cytokines in M1 or M2 Macrophages

**Figure 1:**
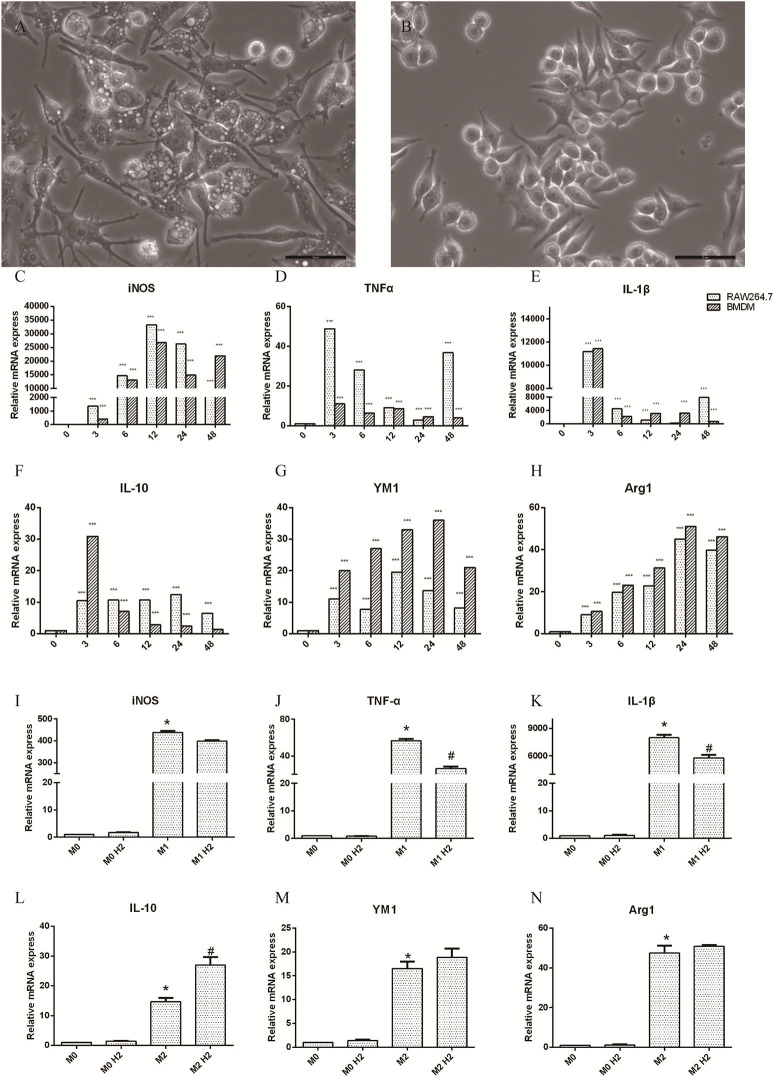
Effects of macrophages on stimulations of macrophages (M1, 20 ng/ml LPS and 50 ng/ml IFN-γ; M2, 20 ng/ml IL-4). **(A)** M1 macrophages (scale bars = 50 μm); **(B)** M2 macrophages (scale bar = 50 μm). The changes of inflammation factors in RAW264.7 and BMDM in different mediums and different times, and mRNA levels of iNOS **(C)**, TNF-α **(D)**, IL-1β **(E)**, IL-10 **(F)**, Arg-1 **(H)**, and protein YM-1 **(G)**. The mRNA levels of in RAW264.7 macrophages with and without hydrogen, iNOS **(I)**, TNF-α **(J)**, IL-1β **(K)**, IL-10 **(L)**, YM-1 **(M)**, Arg-1 **(N)**, ^*^ M1 or M2 vs. M0, ^#^ M1+H_2_ or M2+H_2_ vs. M0 (^*^,^#^ *p* < 0.05, ^**^ *p* < 0.01, ^***^ *p* < 0.001).

RAW264.7 macrophages induced by LPS (20 ng/ml) and IFN-γ (50 ng/ml) at 0, 3, 6, 12, 24, and 48 h were polarized to M1 macrophages, and the axons were small and elongated (Figure 1A). RAW264.7 macrophages induced by IL-4 (20 ng/ml) were polarized to M2 macrophages, which contained stunted axons (Figure 1B). Raw264.7 macrophages and BMDM were cultured with LPS and IFN-γ, and the mRNA levels of inducible nitric oxide synthase (iNOS) (Figure 1C), TNF-α (Figure 1D), and IL-1β (Figure 1E) released by M1 macrophages were significantly upregulated (*p* < 0.001). The mRNA levels of IL-10 (Figure 1F), YM-1 (Figure 1G), and Arg-1 (Figure 1H) produced by M2 macrophages induced by IL-4 were markedly increased as well (*p* < 0.001). These results show that M1 and M2 macrophages were successfully polarized. RAW264.7 macrophages were pretreated with or without hydrogen-rich water and then polarized to M1 or M2 macrophages in different mediums. The mRNA levels of TNF-α (Figure 1J) and IL-1β (Figure 1K) were decreased in the M1+H_2_ group compared to the M1 group (*p* < 0.05). The mRNA levels of IL-10 (Figure 1L) were increased in the M2+H_2_ group compared to the M2 group (*p* < 0.05). However, there is no effects of hydrogen on iNOS (Figure 1I), YM-1 (Figure 1M), Arg-1 (Figure 1N) (^*^, # P < 0.05, ^**^ P < 0.01, ^***^ P < 0.001).

### Expressions of CD16/32 in M1 and CD206 in M2 Macrophages

**Figure 2:**
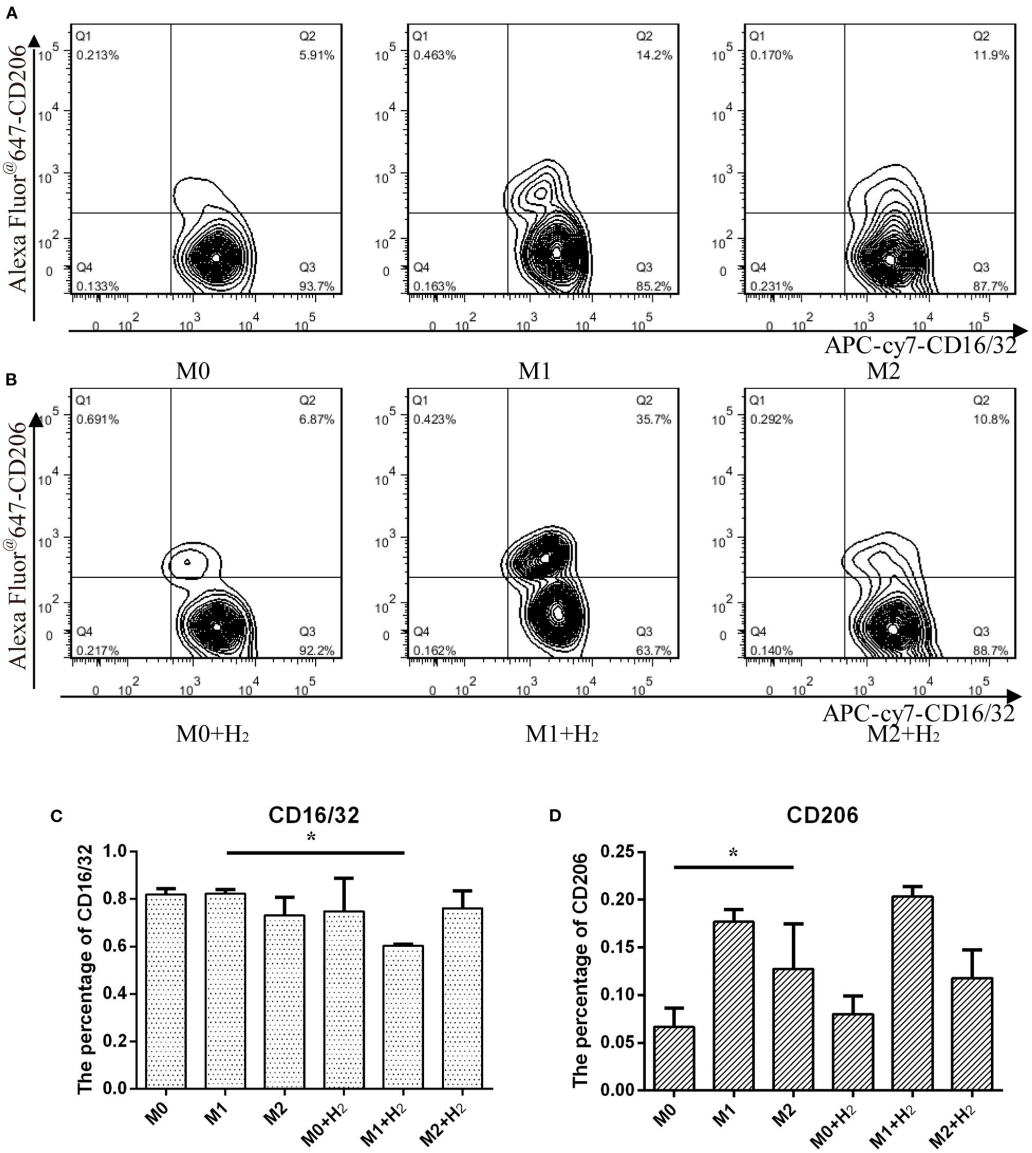
Flow cytometry analysis of macrophages and their markers. Macrophages cultured in normal medium **(A)** and in hydrogen medium **(B)** with different additives, and quantification of CD16/32 **(C)** and CD206 **(D)** represented M1 and M2 macrophages from **(A)** and **(B)**, respectively (^*^p < 0.05, ^**^p < 0.01, ^***^p < 0.001).

In this study, we further investigated the effects of hydrogen on the markers of different macrophages. After treatment with hydrogen for 6 h and then polarizing to M1 or M2 macrophages, respectively, the expression of CD16/32 in M1 macrophages was significantly lower than that of the control cells (*t* = 24.846, *p* < 0.05) (Figures 2A,C). However, hydrogen has no significant influence on the expression of CD206 in M2 macrophages (*t* = 1.284, *p* > 0.05) (Figures 2B,D). The result shows that the expression of CD206 in M2 macrophages was significantly higher than RAW264.7 macrophages (*t* = 3.451, *p* < 0.05), and after stimulation with LPS and IFN-γ, the expression of CD16/32 in M1 macrophages was similar to RAW264.7 macrophages (*t* = 0.976, *p* > 0.05) (^*^, # P < 0.05, ^**^ P < 0.01, ^***^ P < 0.001).

### The Incidence of NEC in Mice Model and Histological Evaluation

**Figure 3:**
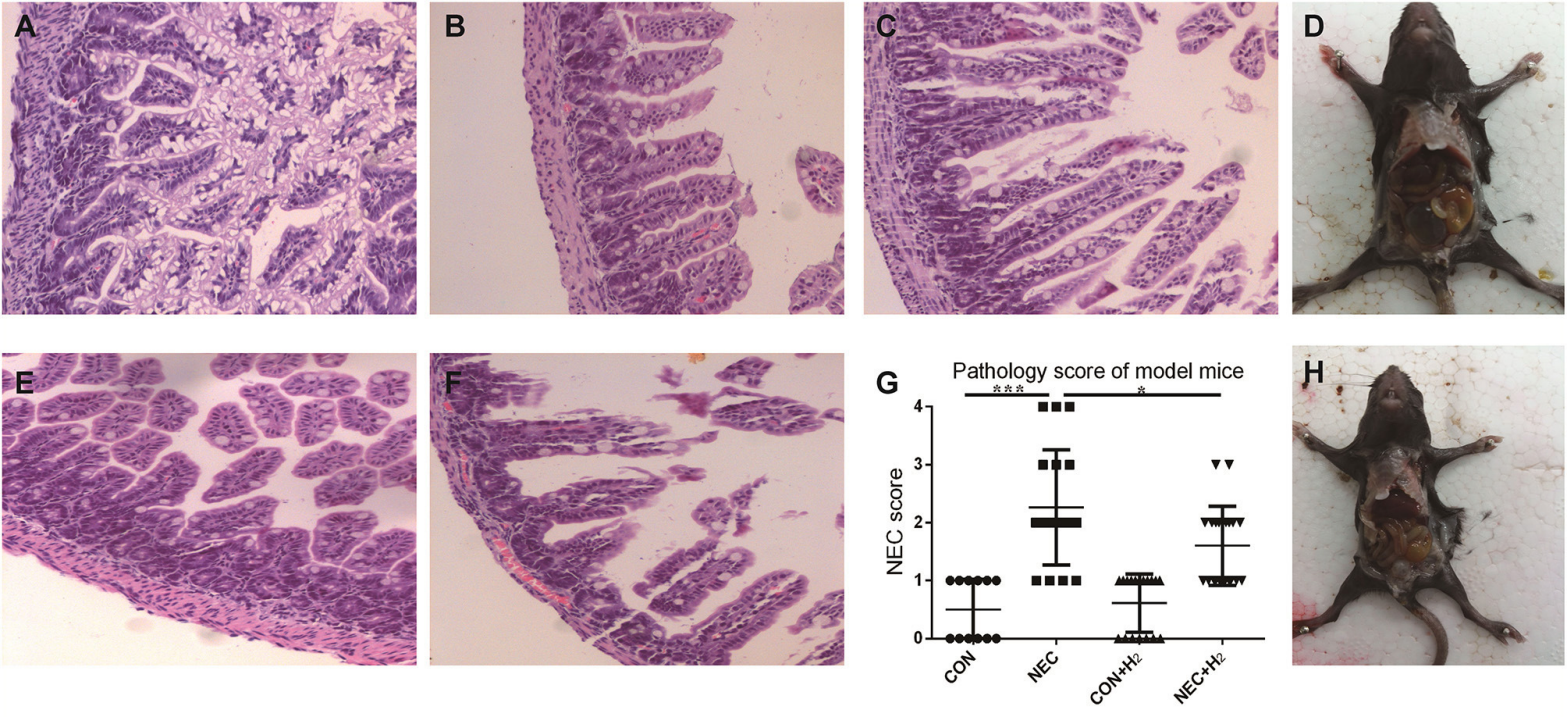
The pathological changes of the different groups. In brief, grade 0-2 **(A–C)**, grade 3 **(E)**, grade 4 **(F)**, **(D,H)** shows the general body of NEC and NEC+ H_2_ mice, respectively. Histological NEC scores of ileum in each group **(G)**. (^*^P < 0.05, ^**^P < 0.01, ^***^P < 0.001).

**Figure 4:**
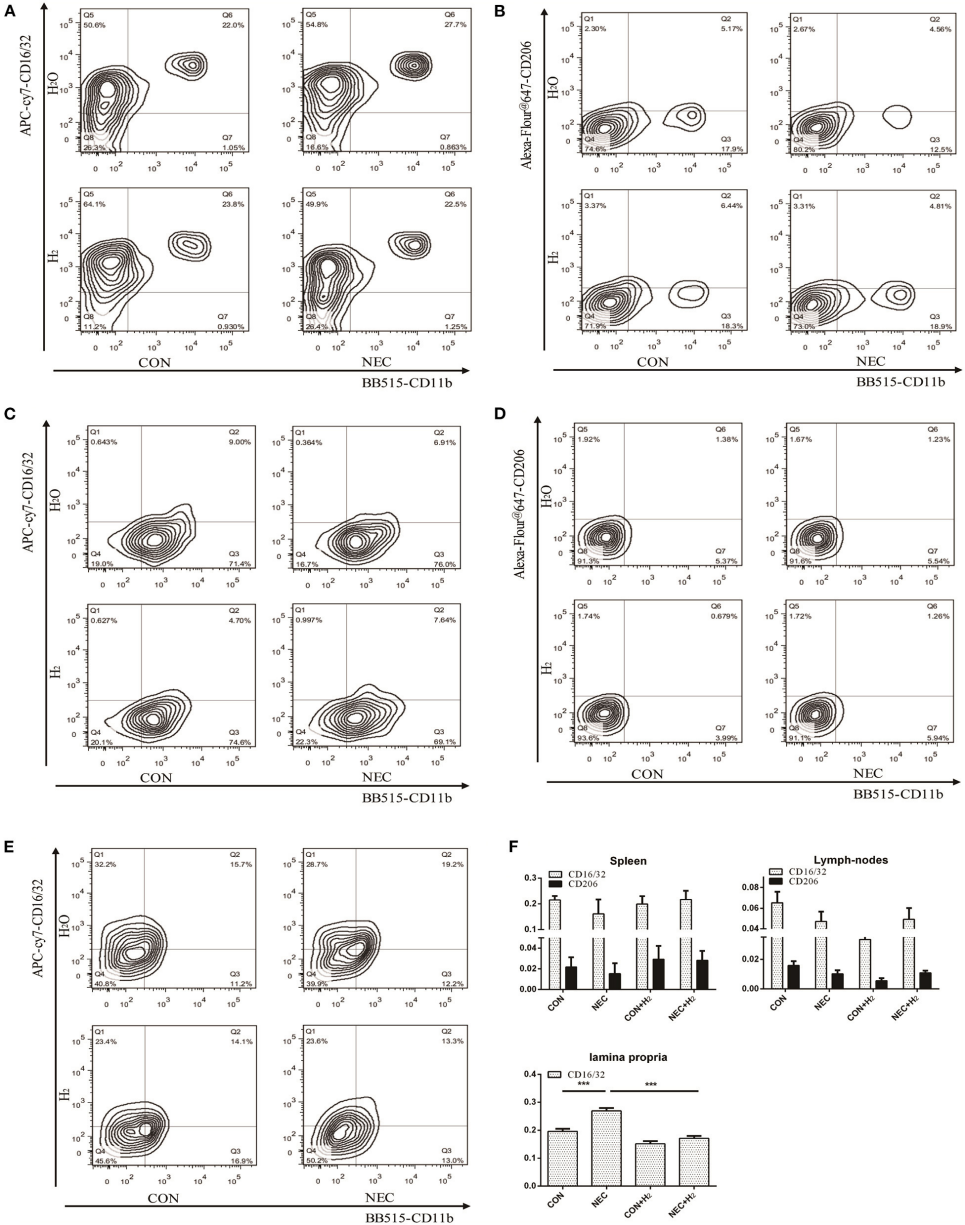
The percentage of macrophages in different tissues. H_2_: fed with hydrogen; H_2_O: fed with water as control. **(A,C,E)** Expression of CD16/32 in spleens, abdominal lymph nodes, and lamina propria, respectively. **(B,D)** Tof HE staining ranginghe expression of CD206 in spleens, abdominal lymph nodes respectively **(B,D)**. **(F)** Quantification of CD16/32 or CD206 macrophages in different tissues obtained from **(A–E)** (^*^P < 0.05, ^**^P < 0.01, ^***^P < 0.001).

The incidence of NEC was 0% in the control group, 52.63% in the NEC group, and 45% in the NEC+ H_2_ group. Figures 3A–C,E,F shows the score of HE staining ranging from 0-4 respectively. Figure 4G shows the histological NEC scores of ileum in each group. Figures 3D,H shows the general body of NEC and NEC+ H_2_ mice, respectively. The mean mucosal injury score in different groups was 0.50 ± 0.51 in the control group, 2.26 ± 0.99 in the NEC group, 0.61 ± 0.50 in the control + H_2_ group, and 1.60 ± 0.68 in the NEC+ H_2_ group (CON vs. NEC: *t* = 6.77, *p* < 0.001; NEC vs. NEC + H_2_, *t* = 2.45, *p* < 0.05; and CON vs. CON + H_2_, *t* = 0.66, *p* > 0.05).

### Analysis of the Percentages of M1 and M2 Macrophages in Different Tissues of Mice

Numerous macrophages with few neutrophils were found in resected tissue samples of neonates affected by NEC. Thus, we attempted to investigate the percentages of macrophages in different tissues of mice model of NEC. Our results showed that they have no significant effect on the percentage of M1 macrophages or M2 macrophages in spleens (Figures 4A,B,F) (NEC vs. CON: CD16/32: *t* = 1.27, *p* > 0.05; CD206: *t* = 0.71, *p* > 0.05) or in abdominal lymph nodes (Figures 4C,D,F) (NEC vs. CON: CD16/32: *t* = 2.06, *p* > 0.05; CD206: *t* = 2.364, *p* > 0.05). However, in lamina propria mucosae of distal ileum, the percentage of M1 macrophages in the NEC group was remarkably higher than that in the control group and the percentage of M1 macrophages was significantly decreased after treatment with hydrogen (Figures 4E,F) (NEC vs. CON, *t* = 9.51, *p* < 0.001; NEC vs. NEC + H_2_, *t* = 12.51, *p* < 0.000) (^*^ P < 0.05, ^**^ P < 0.01, ^***^ P < 0.001).

### Expression of NF-κB p65 in Distal Ileum and M1 Macrophages

**Figure 5:**
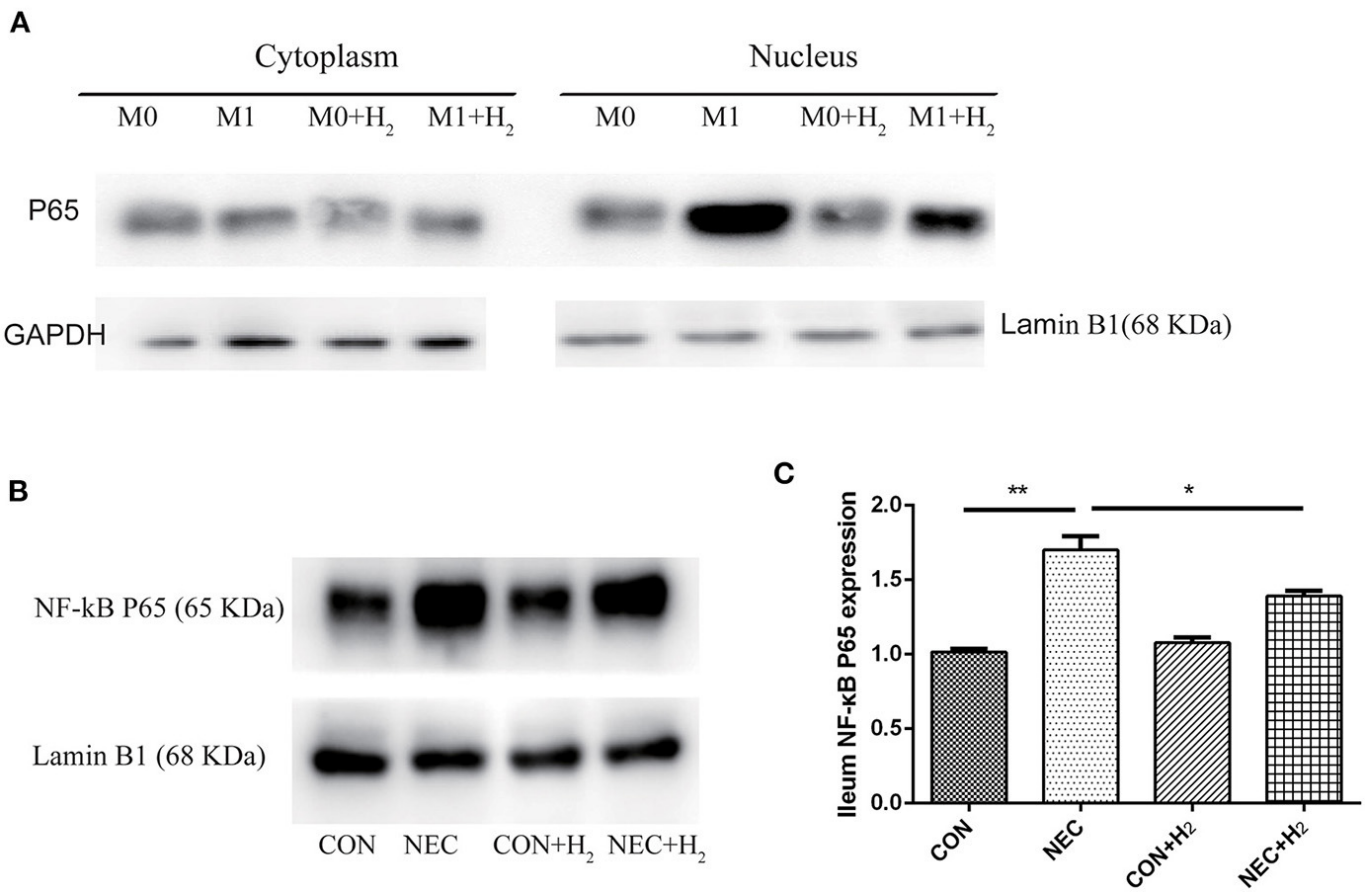
Expression of NF-κB p65 signaling pathway in RAW264.7 and distal ileum of mice. **(A)** Western blotting was undertaken for the expression of NF- κB p65 in the cytoplasm and nucleus of macrophages cell line, **(B)** the expression of NF- κB p65 in distal ileum of NEC mice model. **(C)** Densitometric analysis of the bands of NF-κB p65 in different groups (^*^P < 0.05, ^**^P < 0.01, ^***^P < 0.001).

To understand the effects of hydrogen on polarization of macrophages in M1 macrophages and the mice model of NEC, the level of NF-κB p65 in the nucleus of M1 macrophages (Figure 5A) or distal ileum of mice (Figure 5B) was studied by Western blot analysis. The NF-κB p65 was decreased in M1 macrophage cell nucleus after treatment with hydrogen compared to M1 macrophages (*t* = 16.65, *p* < 0.05) and significantly increased in M1 macrophages after being induced by LPS and IFN-γ. As the same time, we found that the NF-κB p65 was significantly elevated in the NEC group compared with the CON group (1.70 ± 0.09 vs. 1.02 ± 0.02, *p* < 0.01), and decreased in the NEC + H_2_ group compared to the NEC group (Figure 5C) (1.70 ± 0.09 vs. 1.39 ± 0.04, *p* < 0.05).

## Discussion

Macrophages are such important components of the innate immune system, and the functional diversity of the macrophages can be related to their ability on responding to the different micro-environments. With the different stimulated micro-environments, two main phenotypes of macrophages are suggested, M1 macrophages (called classically activated macrophages) and M2 macrophages (called alternatively activated macrophages). M1 macrophages are mainly stimulated by LPS and IFN-γ, and have a key role in host defense against infection. However, M2 macrophages could be stimulated by IL-4 or other factors; they mainly contribute to anti-inflammatory response, and associate with tissue remodeling (9–11, 33). We try to polarize bone marrow-derived macrophages with LPS and IFN-gamma or IL-4 to M1 or M2 in our research; however, with the culture of the time, the less macrophages residue. Afterwards, we only detect the inflammatory cytokines produced by M1 or M2 with rt-PCR, and then we used RAW264.7 cells polarized to M1 or M2 macrophages. The results show that hydrogen has no effect on CD16/32 in RAW264.7 cells; however, the percentage of CD16/32 in M1 macrophages was decreased in the hydrogen group. We supposed that CD16/32 were expressed in most RAW264.7 macrophages, and hydrogen may decrease the expression of CD16/32 in RAW264.7 macrophages. After treatment with hydrogen in RAW264.7 macrophages, the inflammatory cytokines produced by M1 were significantly reduced, and the NF-κB p65 was decreased in M1 macrophage cell nuclei. There may be a connection with the results we found, and that needs to be confirmed further.

NEC is a common gastrointestinal disease, mainly affecting prematurity, and extremely low-birth-weight infants; however, the pathogenesis of NEC is still not fully clear. Hence, in the present study, the NEC model was established to explore the mechanism and therapeutic effects (2, 34, 35). There are many experimental models of NEC available, and researchers may choose different models depending on the purpose of the study. However, we have no idea as to which of the models is truly NEC. In our study, hypoxia, cold stimulation, and artificial formula feeding were used to establish the NEC model, without LPS induction. It was done to exclude the effect of LPS-induced inflammatory stimulation on the outcome. A positive control with LPS may be more appropriate in study design. The older the model animal, the more mature the intestinal development, and the lower incidence of NEC. However, the incidence of NEC in different age groups has not been reported. Pierro et al. found that osmolality of enteral formula had no significant correlation with the incidence of NEC. Therefore, the NEC model was established by hypoxia and cold stimulation and formula feeding (36, 37). The incidence of NEC was 52.63% higher than the control group (*p* < 0.001). By comparing the HE staining score of terminal ileum, it was revealed that the NEC score was decreased from 2.26 ± 0.99 to 1.60 ± 0.68 after treatment with hydrogen. There were no remarkable changes in incidence of NEC between the NEC group and the NEC+ H_2_ group, which may be related to the mice used for the established model, which are too mature even though they are not beyond 14–21 days of age (35). We analyzed the weight of mice during the experiment, in which the results showed that the decrease of body weight in the NEC group was higher than that in the CON group, while there was no significant difference among other groups during the experiment. We supposed hydrogen may not increase the body weight of NEC mice.

We also investigated the percentages of M1 and M2 macrophages in abdominal lymph nodes and spleen in the NEC model; however, the results disclosed that the changes observed among different groups were not statistically significant. Previous studies reported that macrophages in the lamina propria of the intestine plays a key role in the development of an immune response (38–40). Therefore, we assessed the percentage of M1 macrophages in lamina propria. The results showed that expression of CD16/32 M1 macrophages was higher in the NEC group than in the CON group. Moreover, after intervention with hydrogen, the expression of CD16/32 in M1 macrophages was remarkably decreased.

Hydrogen has been shown to have selectively reduced ROS and anti-inflammation by Ohsawa et al. (18). Hydrogen is a new therapeutic medical gas with anti-oxidant, anti-inflammatory, and anti-apoptotic effects on cells and organs, but not applied extensively in clinical practice. Instead of hydrogen molecule, hydrogen-rich saline or water, which is safer and easier to apply, may be more suitable for clinical applications. However, there are many issues that need to be resolved (molecular mechanism, the optimal dose and period, long-term effects, etc.) before the practical clinical application. Our team provides a new method for the role of hydrogen in NEC (17), thus giving hope to solve the previous problems.

There are several limitations in the present study. Firstly, we used mice fed with clean water as a control group, other than bed with their mother together. Secondly, we did not analyze the correlation between the NF-κB signaling pathway and the inflammatory cytokines. We will further analyze the molecular mechanisms of hydrogen regarding polarization of macrophages in NEC.

## Conclusions

In conclusion, the achieved results suggest that M1 macrophages play an important role in the pathogenesis of NEC. Hydrogen protects against intestinal injury through reducing the inflammation factors produced by M1 macrophage *via* preventing M1 macrophage conversion. Hydrogen preventing M1 macrophage conversion may be related to the decrease of NF-κB p65 in the nucleus.

## Data Availability Statement

The datasets presented in this study can be found in online repositories. The names of the repository/repositories and accession number(s) can be found in the article/supplementary material.

## Ethics Statement

The animal study was reviewed and approved by and the experimental protocols were performed according to the Ethical Guidelines for the Use of Animals in Research, and this study was approved by the Animal Care Committee of the Children's Hospital of Shanghai.

## Author Contributions

SY and JS contributed to the project conception and design. SY and ZG collected the data and prepared the article. XW and JZ observed the histological slides. QS and ZL interpreted the data and critically revised and approved the final article. All authors contributed to the article and approved the submitted version.

## Funding

National Natural Science Foundation of China (Award Number: 81370743), Recipient: Zhibao LV. 2. Interdisciplinary Funding of Medicine and Engineering obtained from Shanghai Jiao Tong University (Award Number: YG2015ZD13), Recipient: Zhibao LV. 3. Shanghai Natural Science Foundation (Award Number: 17ZR1423100), Recipient: Zhimei Gao.

## Conflict of Interest

The authors declare that the research was conducted in the absence of any commercial or financial relationships that could be construed as a potential conflict of interest.

## Publisher's Note

All claims expressed in this article are solely those of the authors and do not necessarily represent those of their affiliated organizations, or those of the publisher, the editors and the reviewers. Any product that may be evaluated in this article, or claim that may be made by its manufacturer, is not guaranteed or endorsed by the publisher.
